# Metabolomics Approach for Validation of Self-Reported Ibuprofen and Acetaminophen Use

**DOI:** 10.3390/metabo8040055

**Published:** 2018-09-21

**Authors:** Kristine K. Dennis, Brian D. Carter, Susan M. Gapstur, Victoria L. Stevens

**Affiliations:** 1 Nutrition and Health Sciences, Laney Graduate School, Emory University 615 Michael Street, Atlanta, GA 30322, USA; kkdenni@emory.edu; 2 Behavioral and Epidemiology Research Group, American Cancer Society, 250 Williams Street, NW, Atlanta, GA 30303, USA; brian.carter@cancer.org (B.D.C.); susan.gapstur@cancer.org (S.M.G.)

**Keywords:** metabolomics, acetaminophen, ibuprofen, analgesics, molecular epidemiology

## Abstract

Over-the-counter analgesic use is common and is typically assessed through self-report; therefore, it is subject to misclassification. Detection of drug metabolites in biofluids offers a viable tool for validating self-reported analgesic use. Thus, the aim of this study was to determine the utility of a metabolomics approach for the validation of acetaminophen and ibuprofen use in blood samples. Untargeted mass spectrometry-based metabolomics analysis was conducted in serum samples from 1547 women and plasma samples from 556 men. The presence of two metabolites each for acetaminophen and ibuprofen at levels at or above a defined cutoff value was used to determine concordance with self-reported use. For acetaminophen use based on the presence of both acetaminophen and acetamidophenylglucuronide, concordance was 98.5–100% among individuals reporting use today, and 79.8–91.4% for those reporting never or rare use. Ibuprofen use based on the presence of both carboxyibuprofen and hydroxyibuprofen resulted in concordance of 51.3–52.5% for individuals reporting use today and 99.4–100% for those reporting never or rare use. Our findings suggest that an untargeted metabolomics approach in blood samples may be useful for validating self-reported acetaminophen use. However, this approach appears unlikely to be suitable for validating ibuprofen use.

## 1. Introduction

Use of over-the-counter (OTC) analgesics is common in the United States, both sporadically for acute pain relief and regularly for chronic pain treatment or for some potential health benefit [1,2]. The most common non-prescription pain medications include non-steroidal anti-inflammatory drugs (NSAIDs) such as aspirin and ibuprofen, and non-NSAIDs such as acetaminophen. The potential health benefits linked to OTC analgesic use include reduced risk of breast cancer, colorectal cancer, malignant melanoma [3,4,5], Alzheimer’s disease [6] and heart attacks [7]. However, use of OTC painkillers can result in serious adverse health outcomes, including gastrointestinal bleeding from NSAIDs [8], and toxicity leading to liver failure and potential death from acetaminophen overdose [9]. Because of the high prevalence of use and array of associated health effects, OTC analgesic use is assessed in many epidemiologic studies.

OTC analgesic use is typically self-reported, and therefore, vulnerable to misclassification. Unlike prescription drugs, where cross-referencing with prescription databases, insurance claims, or medical records [10,11,12] can be used to validate self-report, there are no records that can be used to determine the accuracy of OTC analgesic self-reports. Strategies such as home visit inventories and in-depth interviews have been used to validate telephone-based and questionnaire-based self-reported OTC responses, and have reported 86–100% concordance [13,14]. However, limitations of these studies include small sample sizes, unique study populations (e.g., patients undergoing chemotherapy) for which results may not be broadly applicable, and time-intensive cross-validation techniques (i.e. telephone-based interviewing) which may not be feasible in large cohort studies [13,14]. Another strategy for validation is to measure either the drug itself or its metabolites in biospecimens from study participants. This has been done successfully with blood or urinary cotinine to validate self-reported cigarette smoking [15]. However, while analytical methods to measure levels of common OTC analgesics and their metabolites have been developed [16,17,18,19,20], they have not been used for validation purposes in epidemiologic studies. OTC drug metabolites are often measured during untargeted metabolomics analyses, and they could be used to validate self-reported analgesic use. To date, only one study has been published in which such a metabolomics approach was tested. The INTERMAP Research Group used nuclear magnetic resonance spectroscopy (NMR) metabolomic profiles of two 24-hour urine samples [21] to identify spectra corresponding to acetaminophen and ibuprofen metabolites [22]. Results from this study suggest that metabolomics profiling may be feasible to validate self-reported use of these drugs in urine samples. 

In this study, we tested the utility of an untargeted mass spectrometry-based metabolomics approach which detects drug-related metabolites to validate self-reported acetaminophen and ibuprofen use in serum samples from women and plasma samples from men from the Cancer Prevention Study-II (CPS-II) Nutrition Cohort. As blood samples are more commonly collected than urine in epidemiologic studies, understanding the utility of the approach in serum and plasma samples could facilitate future applications in other cohorts. 

## 2. Results 

Participants in this study were elderly (average age of women was 68 years and men was 70 years), predominantly white, and well-educated. Women had higher acetaminophen use today or yesterday (16% in women versus 10% in men), and a larger proportion of men reported never or rare use (59% in women versus 69% in men) (Table 1). Ibuprofen use-patterns were similar in women and men, with about 16% reporting use today or yesterday, and slightly more men reporting never or rare use (59% women versus 64% men) (Table 1).

### 2.1. Acetaminophen Metabolites and Self-Reported Acetaminophen Use

**Figure 1:**
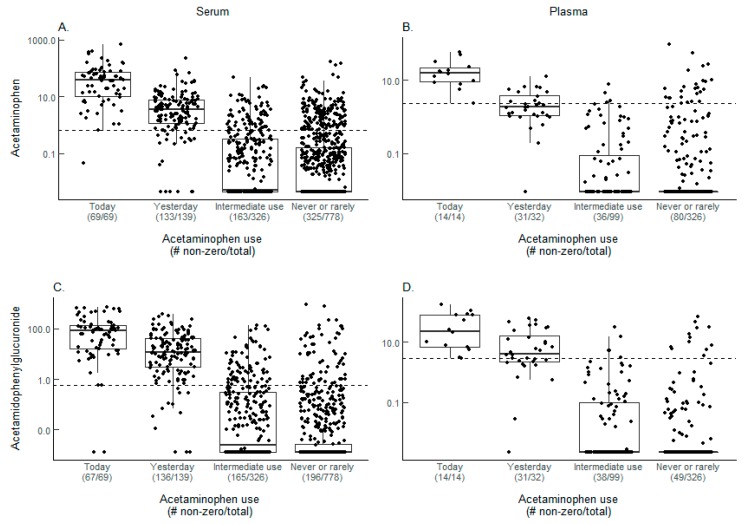
Acetaminophen metabolite levels. Box plots of acetaminophen metabolite levels from serum and plasma samples in log10 scale with undetected (zero) values adjusted to 80% of the minimum value detected for visualization purposes for (**A**,**B**) acetaminophen and (**C**,**D**) acetamidophenylglucuronide. The cutoff levels used to define use for each metabolite are represented by the dashed lines and are (**A**) 0.6578, (**B**) 2.369, (**C**) 0.5633, and (**D**) 2.9350. The number of samples with metabolites detected and the total number of samples analyzed are displayed beneath each self-reported use category. Because the metabolite levels are relative rather than absolute, no units are assigned to the levels.

Both acetaminophen and acetamidophenylglucuronide were above the cutoff level for most of the today user serum (women, Figure 1A,C) and plasma (men, Figure 1B,D) samples and the yesterday user serum samples. However, acetaminophen levels were more evenly split above and below the cutoff level for yesterday user plasma samples. Among never/rare users, many of the detected metabolites were above the cutoff level in both sample types but a higher percentage of the serum samples had detectable metabolites than the plasma samples. 

The acetaminophen metabolite results for serum (women) and plasma (men) are summarized in Table 1. The majority of today users (98.5% in serum and 100% in plasma) had one or both metabolites above the defined cutoff levels, which are the concordance levels for these groups when the presence any metabolite is used to indicate acetaminophen use. For yesterday users, the concordance in serum samples (93.5%) was much higher than in plasma samples (59.4%). Among never/rare users, 79.8% of the serum samples and 91.4% of the plasma samples had neither metabolite. The percent of samples from never/rare users with both metabolites in serum (6.3%) was slightly higher than in plasma (2.8%). For never/rare users, requiring the absence of both metabolites to indicate no acetaminophen use results in a concordance of 79.8% and 91.4%, in serum and plasma samples, respectively. 

Because acetaminophen is a component of many medications, the possibility that the acetaminophen and/or acetamidophenylglucuronide in samples from never/rare users came from either cold medications or prescription pain medication (PPM) was investigated. Of the 157 women (serum) who reported never/rare use of acetaminophen and had at least one metabolite at or above the cutoff level, 19 reported taking cold or PPM either today or yesterday, and an additional 9 took one of these either in the last week or more than a week ago. For the 28 men (plasma) in this group, 5 took one of these medications either today or yesterday and 6 more took them sometime later. Thus, an alternative source of acetaminophen may account for up to 28 or 17.8% of the women (serum) and 11 or 39.3% of the men (plasma) who reported never/rare use having metabolites in their samples.

### 2.2. Ibuprofen Metabolites and Self-Reported Ibuprofen Use

**Figure 2:**
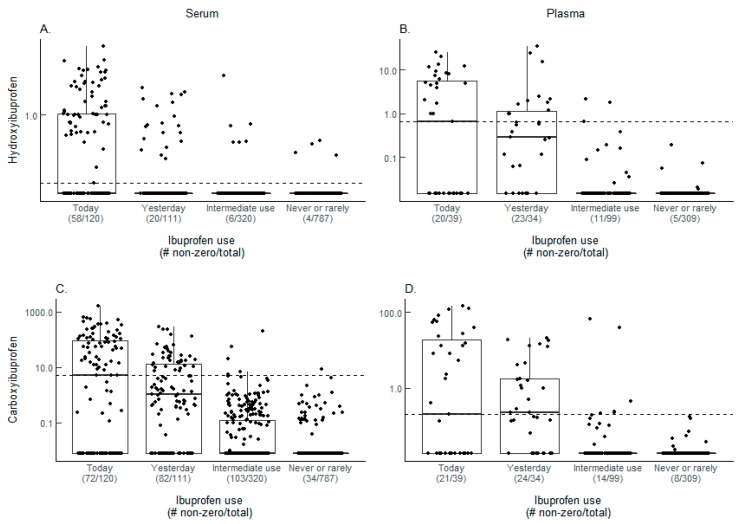
Ibuprofen metabolite levels. Box plots of ibuprofen metabolite levels from serum (women) and plasma (men) samples in log10 scale with undetected (zero) values adjusted to 80% of the minimum value detected for visualization purposes for (**A**,**B**) hydroxyibuprofen and (**C**,**D**) carboxyibuprofen. The cutoff levels used to define use for each metabolite are represented by the dashed lines and are (**A**) 0.2450, (**B**) 0.6487, (**C**) 4.9578, and (**D**) 0.2008. The number of samples with metabolites detected and the total number of samples analyzed are displayed beneath each self-reported use category.

Hydroxyibuprofen and carboxyibuprofen detection was similar among today users in serum (women, Figure 2A,C) and plasma (men, Figure 2B,D), with about half of the participants showing detectable metabolites at or above the cutoff. Only a few serum and plasma samples from never/rare users had detectable levels of one or both metabolites, with most below the cutoff. 

A summary of the ibuprofen metabolite results in serum and plasma is in Table 1. The percentage of samples with both ibuprofen metabolites decreased substantially between today and yesterday users in both serum and plasma. Requiring the presence of at least one metabolite to indicate use results in concordances of 52.5% and 51.3% among today users and 36.0% and 55.9% among yesterday users, in serum and plasma respectively. For never/rare users, concordance as indicated by absence of both metabolites results in 99.4% (serum) and 100% (plasma) concordance levels.

## 3. Discussion

The approach used in this study to investigate the utility of an untargeted metabolomics approach for validation of self-reported acetaminophen and ibuprofen use was modeled, in part, after that used to validate smoking status with nicotine metabolites. However, whereas most smoking studies use only a single metabolite, cotinine, we considered two metabolites for each of the OTC analgesics examined. Requiring the presence of one or two metabolites to define use leads to only those with no metabolites present being defined as nonusers. This criteria for use can lead to higher concordance among self-reported users, particularly yesterday users. Requiring neither metabolite to be present in never/rare users leads to lower concordance among never/rare users, particularly for acetaminophen detection in serum samples. Whether using one or two metabolites provides more accurate results for indicating drug use is not clear based on our findings, and should be investigated further. However, based on the documented half-lives of specific metabolism for these drugs, the definitions used for concordance indicators for today and never/rare users are in line with what would be biologically expected in a general population. 

The concordance between self-reported acetaminophen use today and the presence of one or both metabolites above the cutoff level was 98.5% in women (serum) and 100% in men (plasma), suggesting that metabolomic assessment may be useful for confirming use of this medication within the past 24 h. This concordance was similar to that found when home inventory was compared to self-reported acetaminophen use [13]. Our approach found slightly lower concordance between the absence of two metabolites and never/rare use. There were substantial differences in the percentage with one or both acetaminophen metabolites above the cutoff level between serum and plasma samples from yesterday users. The higher detection in serum samples (82.7% with two metabolites and 93.5% with at least one metabolite) than in plasma samples (34.4% with two metabolites and 59.4% with at least one metabolite) suggests that the clearance or technical detection of the acetaminophen metabolites may differ in these two blood fractions. Although metabolomics data from serum and plasma samples is generally similar, sample extraction (e.g., type of coagulant used), incubation time, and matrix effects can impact detection of metabolites of interest [23,24,25]. 

Use of cold medicine or PPM may account for the presence of one or both acetaminophen metabolites for up to 28 of the women (serum) and 11 of the men (plasma). However, we do not have specific information about the type, and therefore, the ingredients of the cold medicines or PPMs used. For the remainder, the presence of the metabolites could indicate incorrect self-reporting. This seems to be a reasonable explanation for the men among whom 17 (5.2%) would have incorrectly reported never/rare use. However, that 129 (16.6%) women would misreport their acetaminophen use is more than seems likely. Alternatively, it could be that these metabolites persist for a longer time in some people, depending on individual factors such as chronic liver diseases that impact detoxification. However, only a few of the never/rare users with acetaminophen metabolites had liver disease, suggesting that this does not account for the persistence of these metabolites in most cases. Even though the half-life for acetaminophen is 2–3 h for acetaminophen and about 3 h for acetamidophenylglucuronide [26], the persistence of these metabolites in other than today users suggests that our metabolomics analysis can detect very low levels of these compounds, and/or the metabolites are bound or sequestered in some way that allows them to remain in circulation at levels below the detection limits of conventional methodology. Other factors that may contribute to variability in the longevity of the acetaminophen metabolites are the influence of age on acetaminophen metabolism, which is somewhat reduced among these elderly adults [27], and differences in metabolism and elimination due to genetic and gut microbiome variability [28,29,30]. 

In contrast with our acetaminophen results, the concordance between self-reported ibuprofen use and metabolite presence was low for today and yesterday users. Even if only today users were considered, either one or both metabolites were detected in only about half of the serum and plasma samples. On the other hand, the concordance between self-report and metabolites for never/rare users was excellent. With a nonuser definition based on the absence of both metabolites, 99.4% of women (serum samples) and all men (plasma samples) were concordant. However, given the limited ability to detect very recent use, these findings suggest that untargeted metabolomics from blood samples are unlikely to be useful for validation of self-reported ibuprofen use.

Similar to acetaminophen (half-life is 2–3 h), the half-life of ibuprofen is 1.5 to 2 h [31,32]. Consistent with this, our findings suggest that it is rapidly cleared from circulation. The variability in the occurrence of the ibuprofen metabolites among users who took it today could be because the report of today use covers a relatively wide time period, especially for metabolites that are eliminated quickly (e.g., today users can include those who took ibuprofen two hours ago or twenty-three hours ago), possibly preventing accurate detection even among today users. Age-related differences in ibuprofen metabolism, which has been reported in some but not all studies [27], may also contribute to this variability. Finally, the possibility that incorrect self-report of today use occurred in some cases cannot be ruled out.

To our knowledge, the only previous study in which metabolomics data were used to evaluate the validity of self-reported acetaminophen and ibuprofen use was an NMR-based metabolomics study of urine samples from the INTERMAP study. Loo et al. reported a concordance of 81–84% for two separate Caucasian populations using a definition for concordance which combined both analgesics, and grouped nonuser and user results [22]. Combining our results for both drugs and today and never/rare users results in 87.7% concordance among women (serum) and 93.2% among men (plasma). Loo et al. also reported a prevalence of underreporting (metabolite detection when self-report was no use) of 15–17% and underdetection (no metabolite detection when self-report was use) of 1%. In our study, considering anyone among the never/rare users of either analgesic with one or both metabolites as contributing to underreporting yields a prevalence of 10.4% among women (serum) and 4.4% among men (plasma). For underdetection among today users based on the absence of both metabolites and combining acetaminophen and ibuprofen results, underdetection would be 30.7% among women (serum) and 35.8% among men (plasma). Thus, our concordance and underreporting are somewhat better than those of the previous study, while our underdetection is worse. This latter result could be due to the fact that we used a blood sample collected sometime after analgesic use while the previous study used a 24-hour urine sample that presumably would collect metabolites immediately after drug use [22].

Our findings demonstrate the potential utility and challenges of using untargeted metabolomics results from blood samples to assess self-reported acetaminophen and ibuprofen use. To our knowledge, this is the first study to use untargeted metabolomics data generated from blood samples to assess self-reported ibuprofen and acetaminophen use. Given the increasing availability of untargeted metabolomics data from epidemiologic studies, there is a significant opportunity to validate self-report data with existing datasets. Future research to better tailor validation approaches with metabolomics data are needed. Including more metabolites, weighting metabolites based on favored metabolism pathways, and obtaining better exposure information would increase the utility of metabolomics data for validation. Assessment of other self-report data may be possible through similar applications of untargeted metabolomics data. 

## 4. Materials and Methods

### 4.1. Study Population

Study participants were drawn from the CPS-II Nutrition Cohort, a prospective study of cancer incidence and mortality established by the American Cancer Society in 1992 [33]. Participants completed an initial baseline questionnaire on demographic, behavioral, environmental, and occupational factors and follow-up questionnaires every two years starting in 1997 to ascertain self-reported cancer incidence and update exposure status. Between 1998 and 2001, participants were invited to provide a blood sample and complete a brief questionnaire about specific risk factors such as medication use, smoking status, and other factors related to the blood collection. All blood samples were acquired at medical facilities in the participant’s community using standardized collection protocols. Plasma samples were derived from blood collected in EDTA-containing vacutainers, while serum samples were from blood collected in serum separator tubes. Blood samples were shipped overnight to a central repository for fractionation and long-term storage in liquid nitrogen freezers. Informed consent was obtained from all individual participants included in this study. All aspects of the CPS-II Nutrition Cohort were conducted in accordance with the Declaration of Helsinki and were approved by the Institutional Review Board of Emory University (Atlanta, GA, USA, IRB00045780).

This analysis draws from two separate untargeted metabolomics analyses involving participants from the CPS-II Nutrition Cohort. The first analysis included serum samples from 1547 postmenopausal women who were selected for a nested case-control study of breast cancer. Cases were required to be postmenopausal at blood donation and to have a verified invasive breast cancer diagnosis after blood draw. For each case, a single control was matched on age (within 6 months) and race from among the women who were cancer-free at the time of diagnosis of the matched case. The second analysis included plasma samples from 556 men who were selected for a case-cohort study of advanced and lethal prostate cancer. Cases were required to have either a verified diagnosis of advanced prostate cancer (American Joint Committee on Cancer stage 3 or 4) after blood donation or to have died from prostate cancer. Cohort samples were randomly selected from among the available male participants [34].

### 4.2. Questionnaire Data 

The brief questionnaire completed at the time of blood draw asked about use of various medications, including ibuprofen and acetaminophen. Participants were asked “When was the last time that you took this?” for acetaminophen and ibuprofen. Possible responses were “today”, “yesterday”, “in the last week”, “more than one week ago” and “never or rarely”. For the analysis, use “in the last week” and “more than one week ago” were combined into a single category called “intermediate”. Similar information regarding cold medicine and PPM was also ascertained but no specific information about the type of cold medicine or PPM was collected. 

For this analysis, participants (141 women and 61 men) were excluded because information for both ibuprofen and acetaminophen use was missing. Of the 1406 remaining women, participants missing only self-report data for ibuprofen (N = 68) and acetaminophen (N = 94) were excluded in their respective sub-analyses for metabolite detection and self-reported use status. Of the 495 remaining men, participants missing only self-report data for ibuprofen (N = 14) or acetaminophen (N = 24) were excluded in their respective sub-analyses for metabolite detection and self-reported use status.

### 4.3. Metabolomics Analysis

Metabolomics analysis was completed by Metabolon, Inc. (Durham, NC, USA) using the previously-described methodology [35]. Samples were treated with methanol, aliquoted, and then analyzed using four platforms to provide the broadest coverage of metabolites: two separate reversed phase (RP)/ultra-high-performance liquid chromatography-tandem mass spectroscopy (UPLC-MS/MS) methods with positive ion mode electrospray ionization (ESI), one RP/UPLC-MS/MS method with negative ion mode ESI and one hydrophobic interaction chromatography (HILIC)/UPLC-MS/MS with negative ion mode ESI. The metabolites further analyzed in this study (acetaminophen, acetamidophenylglucuronide, carboxyibuprofen, and hydroxyibuprofen) were from the untargeted metabolomics data generated by these four platforms.

A reference library of over 3300 chemical standards was used for comparison to ion features of the study samples for metabolite identification. Peaks were quantified using area-under-the-curve (AUC), and levels reflect relative rather than absolute amounts. Day-to-day variation was corrected for by setting median values of each compound to 1 for each run-day and normalizing each data point proportionately. Thus, the level for each metabolite was the AUC/median AUC for the day. Any missing values were considered to reflect amounts below the level of detection. However, for the construction of boxplots showing the distribution of metabolite levels on the log scale, the level of the undetected metabolites was set to 80% of the minimum value detected. The reliability of the metabolomics analyses was evaluated using replicate quality control samples run with the study samples. For the serum metabolites, the median intraclass correlation coefficient (ICC) was 0.90 with an interquartile range (IQR) of 0.74 to 0.96 and for the plasma metabolites, the median ICC was 0.89 with an IQR of 0.66 to 0.97. The ICCs for the four acetaminophen and ibuprofen metabolites was 0.93 across both sample types, with the exception of carboxyibuprofen in plasma samples (ICC = 0.67). 

**Figure 3:**
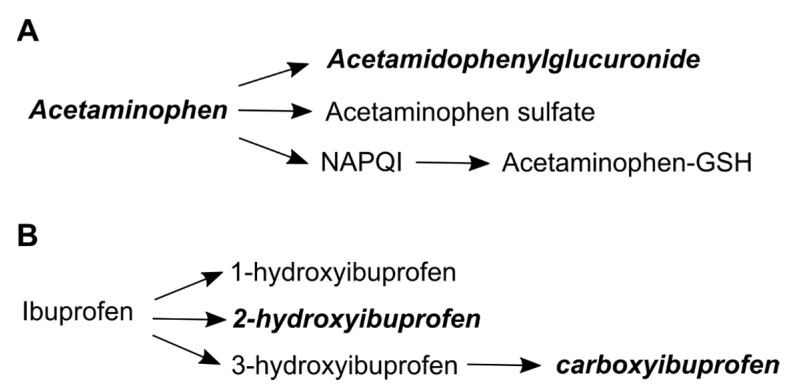
Acetaminophen and ibuprofen metabolism. Initial metabolism steps for acetaminophen (**A**), and ibuprofen (**B**). Bolded metabolites were used as indicators to assess use. Abbreviations: NAPQI is N-acetyl-p-benzoquinone and GSH is glutathione.

The relevant metabolic pathways for acetaminophen and ibuprofen are shown in Figure 3. Acetaminophen use was assessed with acetaminophen (HMDB01859), the parent compound, and acetamidophenylglucuronide (HMDB10316), a primary elimination metabolite [32,35]. Ibuprofen use was assessed using two primary ibuprofen metabolites, hydroxyibuprofen (HMBD60920) and carboxyibuprofen (HMDB60564) [36]. The Metabolomics Standard Initiative identification level for all four of these metabolites was level 1 [37].

### 4.4. Data Analysis

All data analysis was completed in R statistical language version 3.4.2 (2017-09-28). Following the approach commonly used for assessing smoking status using cotinine levels [38], cutoff levels for each metabolite were defined as the lowest level found in today users with detectable levels of both analgesic metabolites of interest (e.g., acetaminophen and acetamidophenylglucuronide for acetaminophen use). The cutoff levels for each metabolite were then applied to establish concordance between self-reported analgesic use and metabolite detection. 

Self-reported use and metabolite profiles were considered concordant when either today and/or yesterday use (as indicated) was reported and one or both metabolites of interest were present at or above the defined cutoff levels, or when use was reported to be never/rare and neither metabolite was present at or above the cutoff levels. Other combinations of reported use and metabolite levels (today or yesterday use and no metabolites; never/rare use and one or two metabolites) were considered non-concordant.

## Figures and Tables

**Table 1:** Comparison of self-reported acetaminophen and ibuprofen use and detection of specific metabolites in plasma and serum samples.

Drug Metabolite(s) Detected ^a^	Serum				Plasma			
	Self-Reported Drug Use				Self-Reported Drug Use			
	Today	Yesterday	Intermediate ^b^	Never/Rare	Today	Yesterday	Intermediate ^b^	Never/Rare
	N (%)	N (%)	N (%)	N (%)	N (%)	N (%)	N (%)	N (%)
Acetaminophen	69	139	326	778	14	32	99	326
Acetaminophen + Acetamidophenylglucuronide	67 (97.1)	115 (82.7)	41 (12.6)	49 (6.3)	14 (100.0)	11 (34.4)	1 (1.0)	9 (2.8)
Acetaminophen only	1 (1.4)	3 (2.2)	25 (7.7)	94 (12.1)	0 (0)	0 (0)	3 (3.0)	16 (4.9)
Acetamidophenylglucuronide only	0 (0)	12 (8.6)	25 (7.7)	14 (1.8)	0 (0)	8 (25.0)	4 (4.0)	3 (0.9)
None	1 (1.4)	9 (6.5)	235 (72.1)	621 (79.8)	0 (0)	13 (40.6)	91 (91.9)	298 (91.4)
Ibuprofen	120	111	320	787	39	34	99	309
Carboxyibuprofen + Hydroxyibuprofen	57 (47.5)	17 (15.3)	3 (0.9)	0 (0)	20 (51.3)	10 (29.4)	2 (2.0)	0 (0)
Hydroxyibuprofen Only	1 (0.8)	3 (2.7)	3 (0.9)	4 (0.5)	0 (0)	1 (2.9)	0 (0)	0 (0)
Carboxyibuprofen Only	5 (4.2)	20 (18.0)	2 (0.6)	1 (0.1)	0 (0)	8 (23.5)	4 (4.0)	0 (0)
None	57 (47.5)	71 (64.0)	312 (97.5)	782 (99.4)	19 (48.7)	15 (44.1)	93 (93.9)	309 (100.0)

^a^ Metabolite was considered detected if the value was at or above the cutoff value for the indicated metabolite. Missing self-report acetaminophen use responses for serum (N = 94) and plasma (N = 24), and self-reported ibuprofen use responses for serum (N = 68) and plasma (N = 14). ^b^ Intermediate users include those reporting use “in the last week” and “more than one week ago”.
